# Ten Years of Abramson Experience in Patients With Pectus Carinatum

**DOI:** 10.1093/icvts/ivaf268

**Published:** 2025-11-08

**Authors:** Volkan Karaçam, Fatma Mutlu, Emrah Karci, İlkay Kaya, Aydın Şanli

**Affiliations:** Department of Thoracic Surgery, Dokuz Eylul University, Izmir, Turkey; Department of Thoracic Surgery, Dokuz Eylul University, Izmir, Turkey; Department of Thoracic Surgery, Dokuz Eylul University, Izmir, Turkey; Department of Thoracic Surgery, Dokuz Eylul University, Izmir, Turkey; Department of Thoracic Surgery, Dokuz Eylul University, Izmir, Turkey

**Keywords:** pectus carinatum, Abramson procedure, Single Step Questionnaire (SSQ)

## Abstract

**Objectives:**

Pectus carinatum is the second most common congenital chest wall deformity. While open thoracic reconstruction surgeries like the Ravitch procedure remain a treatment option, minimally invasive techniques such as the Abramson procedure are increasingly preferred. This study presents our clinical experience with the Abramson procedure for pectus carinatum.

**Materials and methods:**

A retrospective review was conducted on 86 patients who underwent the Abramson procedure in the Department of Thoracic Surgery at Dokuz Eylul University between 2011 and 2021. Data were collected on age, gender, the number of bars and stabilizers used, postoperative complications, associated anomalies, hospitalization, and bar removal time. All patients completed the newly developed Single Step Questionnaire (SSQ) by Krasopoulos to assess their satisfaction.

**Results:**

All patients underwent bilateral 2-incision procedures using 1 bar and 2 stabilizers. Early postoperative complications were absent in 94.2% (*n* = 81) of patients, while late complications were not recorded in 91.8% of patients (*n* = 79). Associated anomalies included scoliosis (*n* = 5), kyphosis (*n* = 1), and kyphoscoliosis (*n* = 1). The mean hospitalization was 3.7 days. Bars were removed in 75 patients (87.2%), most commonly between the 25th and 36th months postoperatively. The mean satisfaction score was 88.56.

**Conclusions:**

The Abramson technique is a safe, minimally invasive option for selected pectus carinatum cases, offering shorter hospital stays and better aesthetic outcomes compared to open techniques. This study is the first to report satisfaction survey results for the Abramson procedure, highlighting its high patient satisfaction rates.

## Graphical Abstract

**Figure:**
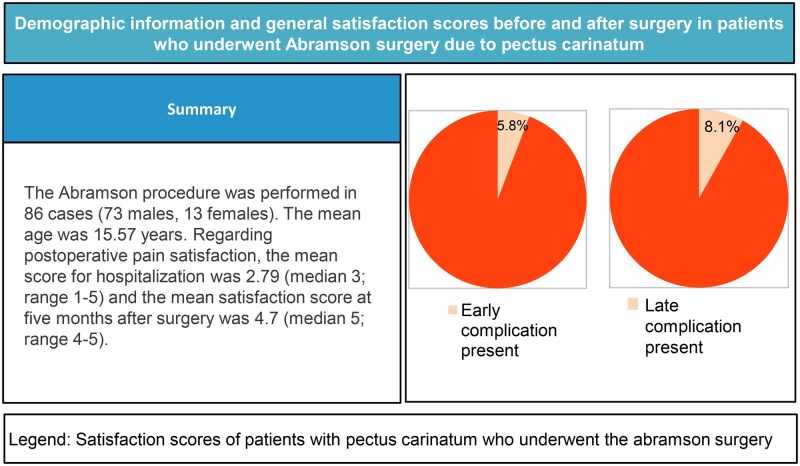

## INTRODUCTION

Pectus carinatum (PC) is a congenital chest wall deformity characterized by anterior protrusion of the sternum and costal cartilages due to abnormal growth, with a prevalence of approximately 0.06%.^1^^,^^2^ It is the second most common chest wall deformity after pectus excavatum (PE), predominantly affecting males with a 4:1 male-to-female ratio.^1^ Although its aetiology is unclear, it is attributed to defective costal cartilage growth.^2^

PC is classified into 3 types based on morphology: chondrogladiolar (most common, involving the lower sternum, symmetric or asymmetric), chondromanubrial (rarer, involving the upper sternum), and mixed forms.^3^^,^^4^

Surgical treatment for PC began in 1911 with open reconstruction and costal cartilage resection.^5–7^ Following Nuss et al’s introduction of a non-resective technique for chest wall repair in 1998, non-resective methods gained popularity.^8^ In 2005, Abramson introduced a minimally invasive technique involving placement of a steel bar over the sternum, secured to the ribs.^9^

This study aims to present our 10-year clinical experience and patient satisfaction outcomes using the Abramson technique for PC deformity.

## MATERIALS AND METHODS

A total of 86 cases who underwent the Abramson procedure at the Department of Thoracic Surgery, Dokuz Eylul University, between 2011 and 2021, were included in the study. Patients older than 12 who were non-compliant with or unresponsive to corset treatment, or who had excessive chest wall rigidity, were included. Patients younger than 12, those who responded to the compression test, those with deformities above the breast level, and those with pectus arcuatum deformity were not considered suitable candidates for the Abramson procedure.

**Figure 1.**
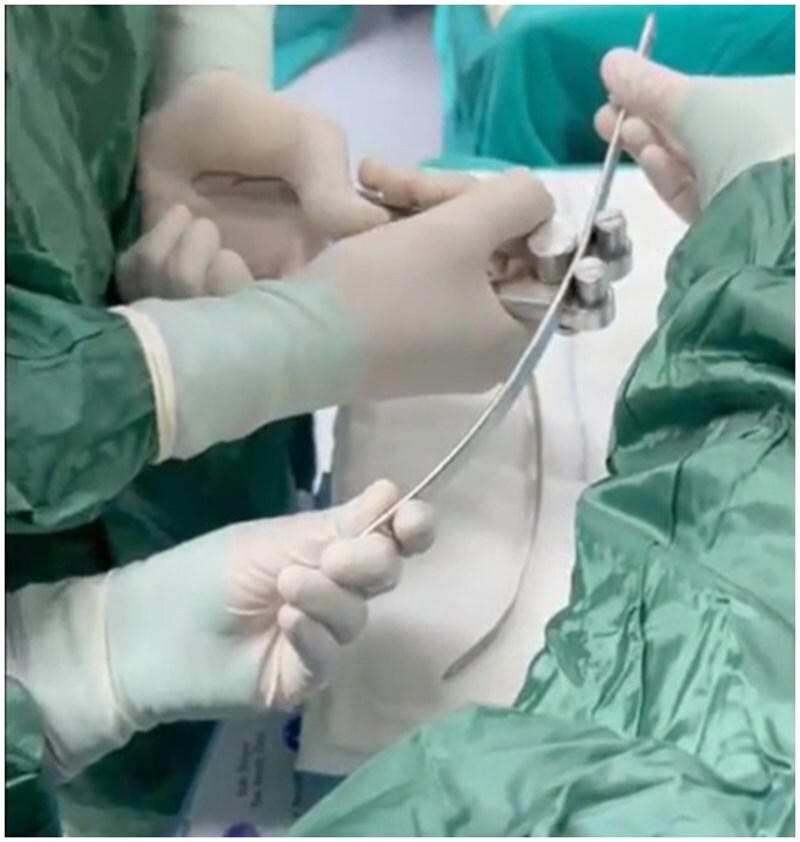
The Steel Bar Is Shaped Using Benders According to the Desired Contour to Be Achieved on the Thoracic Wall.

**Figure 2.**
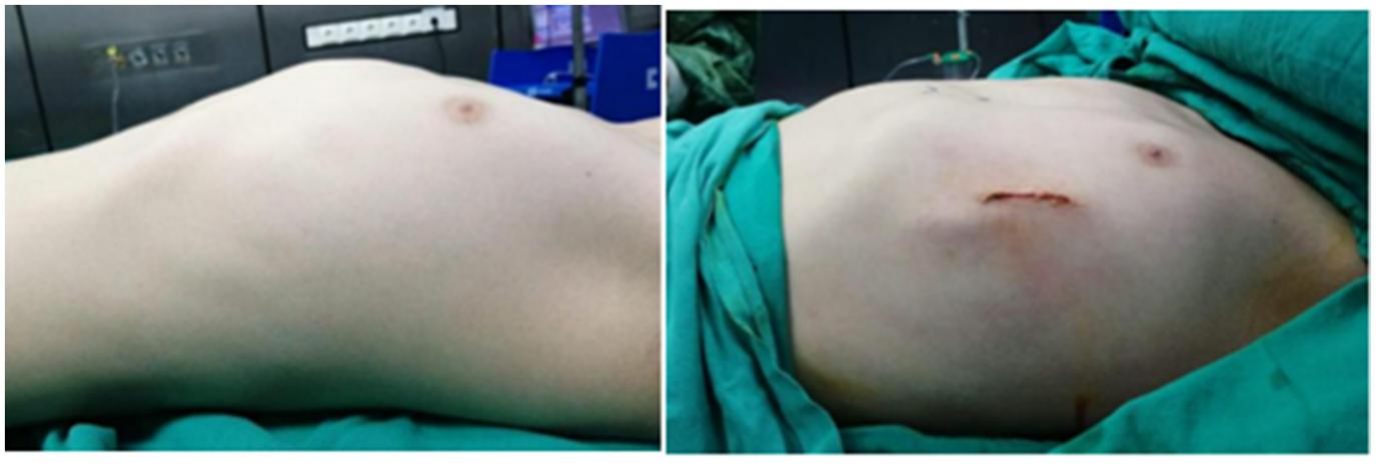
Preoperative and Postoperative Images of a Patient Who Underwent the Abramson Procedure.

In all cases, under endotracheal intubation and general anaesthesia, patients were positioned supine with both arms abducted. The highest point of the deformity was identified. In all patients, bilateral 3 cm incisions were made in the midaxillary line at the level where the bar would be placed. To fix the stabilizers, the skin-subcutaneous tissues and serratus muscle were dissected until reaching the ribs, one intercostal space above and below the planned bar level. The stabilizer was placed in this area. The bar was placed in the tunnel created by dissecting with long clamps between the sternum and pectoral muscles, between both incision lines. Steel wires were passed around 2 consecutive rib levels on both sides for fixation before being threaded through the stabilizer. For this purpose, the ribs are dissected subperiosteally, and a Doyen clamp is passed completely around each rib. An aspiration catheter is attached to the tip of the Doyen clamp, and the clamp is withdrawn. Then, steel sutures are passed through the lumen of the aspiration catheters, and the catheters are withdrawn. This technique allows the wires to be passed around the ribs without damaging the intercostal structures. With the help of a guide, a shaped steel bar was placed over the sternum to provide pressure to the chest. The ends of the bar are attached to the previously placed stabilizers, and compression is applied to the sternum while the stabilizers are firmly fixed to the ribs. Subsequently, bilateral traction is applied to the bars, which are bent with the help of benders and fixed in place using screws. After ensuring haemostasis, the layers are closed in accordance with the anatomical planes (**Figure 1**). A single bar was used in all cases due to its ability to provide sufficient reconstruction (**Figure 2**).

All patients were followed up at 10 days, 1 month, and then at 6-month intervals after the Abramson procedure until the bar was removed. Postoperative follow-ups were performed at 10 days, 1 month, and 1 year after the bar was removed.

The cases included in the study were retrospectively evaluated based on age, gender, number of bars and stabilizers used, number of incisions, early and late postoperative complications, associated anomalies, hospitalization duration, and bar removal time. To assess patient satisfaction, the Single Step Questionnaire (SSQ) was developed by Krasopoulos et al, which allows for scoring of each question as well as calculating an overall score for each patient and can be applied postoperatively by the doctor (**Table 1**). The questionnaires were administered face-to-face during routine clinic visits. For patients who completed the 1-year follow-up after the bar was removed and who were lost to follow-up, patients were contacted and invited to the clinic, and the questionnaires were administered face-to-face. In patients who could not come to the clinic, the questionnaires were administered by a thoracic surgeon via telephone interviews. All statistical analyses were conducted using IBM SPSS Statistics version 19.0 (SPSS Inc., Chicago, IL, United States). Continuous variables, including age, operation time, hospital stay, and SSQ scores, were expressed as mean ± SD, median, minimum, and maximum values. Categorical variables such as gender, presence of complications, bar size, and associated anomalies were presented as frequencies and percentages.

**Table 1.** The New Single Step Questionnaire

1	How was your general health status after surgery?	Much better 5, a little better 4, no change 3, a little worse 2, much worse 1
2	How was your exercise capacity after surgery?	Much better 5, a little better 4, no change 3, a little worse 2, much worse 1
3	How did the appearance of your chest affect your social activity before surgery?	Extremely 5, quite a bit 4, somewhat 3, slightly 2, not at all 1
4	How did the appearance of your chest affect your social activity after surgery?	Not at all 5, slightly 4, undecided 3, quite a bit 2, extremely 1
5	Were you satisfied with the appearance of your chest after surgery?	Very satisfied 5, somewhat satisfied 4, neutral 3, somewhat dissatisfied 2, very dissatisfied 1
6	Were you uncomfortable with the scar?	Not bothered at all 5, slightly bothered 4, undecided 3, a little 2, very bothered 1
7	Did the surgery affect your social life?	Very good effect 5, good effect 4, not effect 3, bad effect 2, very bad effect 1
8	Would you rate your self-esteem/confidence on a scale of 1 to 10 before surgery?	1 to 10 (1 being the lowest, 10 being the highest)
9	Would you rate your self-esteem/confidence on a scale of 1 to 10 after surgery?	1 to 10 (1 being the lowest, 10 being the highest)
10	How was your pain level during the first days post-surgery?	None 5, mild 4, moderate 3, severe 2, very severe 1
11	Did the surgery affect your daily activities during the first 5 months?	Not at all 5, slightly 4, moderately 3, a lot 2, extremely 1
12	How was your pain level 5 months after surgery?	Not at all 5, sometimes 4, slightly when I do not use painkillers 3, slightly when I use painkillers 2, very much 1
13	Did you feel the presence of a metal bar in your body?	Not at all 5, a little 4, undecided 3, quite a lot 2, very much 1
14	How would you rate the final result after surgery?	Fully satisfied 5, satisfied 4, no change 3, somewhat dissatisfied 2, very dissatisfied 1
15	How do you evaluate the change in your chest?	Completely improved 5, improved 4, no change 3, now worse 2, very bad 1
16	Would you have the surgery again?	Yes 10, undecided 5, no 0

To evaluate differences in satisfaction scores (SSQ) between patients with and without complications or additional deformities, normality was assessed using the Shapiro-Wilk test. Since the SSQ scores were not normally distributed, comparisons between groups were performed using the Mann-Whitney *U* test for independent samples. A *P*-value of <.05 was considered statistically significant.

Ethical approval for this study was obtained from the Non-Interventional Ethics Committee of Dokuz Eylul University on October 12, 2022 (Decision No. 2022/32-05).

## RESULTS

A total of 86 patients were included in the study, with a mean age of 15.57 years (median: 15, range: 12-32 years). Of these, 73 were male (84.9%) and 13 were female (15.1%). Although exercise-induced chest pain was reported in some of the operated cases, the primary reason for presentation in the majority of cases was cosmetic concern. Scoliosis was present in 5 patients (5.8%), kyphosis in 1 patient (1.16%), and kyphoscoliosis in 1 patient (1.16%). The Abramson procedure was performed on the patients using bilateral incisions, with 1 bar and 2 stabilizers (1 at each end of the bar) placed bilaterally. The most commonly used bar was 280 mm (22 patients, 25.6%), followed by 300 mm (15 patients, 20.9%). The mean operation time was calculated as 80.3 min (median: 85, range: 40-120 min).

In 94.2% of the patients (*n* = 81), no complications were observed during the early postoperative period (early complications 5.8% [95% CI: 0.9%-10.8%]). However, pneumothorax was reported only in 5 patients (5.8%) during this period. After transfer to the ward, 1 patient required tube thoracostomy, and 2 required pleural drainage catheters. In 2 patients, pneumothorax was resolved with needle aspiration followed by oxygen therapy.

In 79 patients, no late postoperative complications were observed (late complications 8.1% [95% CI: 2.4%-13.9%]). In 1 patient (1.2%), bar displacement was observed 12 months later, requiring corrective surgery. In 2 patients (2.3%), wound debridement was carried out. Although wire breakage is reported as the most common late postoperative complication in the literature, it was observed in only 3 of our patients (3.5%). In 1 patient (1.2%), PE deformity developed 4 years after bar removal (overcorrection), necessitating a Nuss procedure (**Table 2**). In all 3 cases with wire breakage, the breaking occurred after the first year postoperatively. No additional surgical intervention was performed, and the patients were closely followed. After achieving sufficient correction, the bars were removed.

**Table 2.** Late Postoperative Complications

	*n*	%
Bar displacement	1	1.2
Wound dehiscence	2	2.3
Overcorrection	1	1.2
Wire breakage	3	3.5
No complications	79	91.8
Total	86	100.0

In 11 patients (12.8%), the bar remains in place, while 75 patients (87.2%) have had their bar removed. In 54 patients (62.8%), the bar was removed after 36 months, and in 2 (2.3%) patients, it was removed after 60 months (**Table 3**). In 3 cases, the bars were removed before 12 months due to wound infection and bar incompatibility. The length of hospital stay ranged from a minimum of 2 days to a maximum of 6 days. The most common discharge day was postoperative day 4 (28 patients, 32.6%).

**Table 3.** Bar Removal Time

	*n*	%
0-12 months	3	3.5
13-24 months	11	12.8
25-36 months	40	46.5
37-48 months	15	17.4
49-60 months	4	4.7
After 5 years	2	2.3
Bar still in place	11	12.8
Total	86	100.0

The modified Krasopoulos questionnaire, used to assess postoperative patient satisfaction, was administered to all patients, both those who had the bar removed and those who had not. The total score ranged from a minimum of 70 to a maximum of 92, with an average score of 88.56 (**Table 4**). When the group without bar removal was excluded, the mean score was 88.64. The average preoperative self-confidence score of patients was 3.72 (median: 3; min: 1; max: 5), whereas the postoperative self-confidence score increased to 9.4 (median: 9; min: 8; max: 10) (Questions 8 and 9). The average score for postoperative exercise capacity was 4.35 (median: 4; range: 3-5) (Question 2). The average score for satisfaction with the postoperative appearance of the chest wall was 4.72 (median: 5) (Question 5).

**Table 4.** Analysis of Responses to Single Step Questionnaire Questions

	Minimum	Maximum	Mean	Median	SD
Question 1 (1-5 points)	3	5	4.15	4	0.914
Question 2 (1-5 points)	3	5	4.35	4	1.099
Question 3 (1-5 points)	4	5	4.65	4	1.166
Question 4 (1-5 points)	4	5	4.31	4	1.090
Question 5 (1-5 points)	4	5	4.72	5	1.199
Question 6 (1-5 points)	3	5	4.19	4	1.420
Question 7 (1-5 points)	4	5	4.76	5	0.943
Question 8 (1-10 points)	1	5	3.72	3	1.986
Question 9 (1-10 points)	8	10	9.40	9	1.133
Question 10 (1-5 points)	1	5	2.79	3	1.535
Question 11 (1-5 points)	1	5	3.21	3	1.356
Question 12 (1-5 points)	4	5	4.70	5	1.050
Question 13 (1-5 points)	1	5	4.10	4	1.198
Question 14 (1-5 points)	4	5	4.73	5	1.093
Question 15 (1-5 points)	4	5	4.62	5	1.011
Question 16 (0-5-10 points)	5	10	9.76	10	1.142

In response to the question “If you go back, would you have surgery again?” 82 patients (95.3%) answered “yes,” while 4 patients (4.6%) answered “undecided” (Question 16). Among the undecided group, the most common reasons cited were postoperative pain and discomfort from the scar. In terms of postoperative pain satisfaction, the average score for hospitalization was 2.79 (median 3; range 1-5), and for the period 5 months after surgery, the average score was 4.7 (median 5; range 4-5) (Questions 10, 12). For the satisfaction with the surgical scar, the average score was 4.19 (median 4; range 3-5) (Question 6). The relationship between patient satisfaction and the presence of complications, as well as additional deformities, was analysed. No statistically significant differences were observed (*P* > .05).

The overall satisfaction average score of the final outcome was calculated as 4 out of 5 (Question 14).

## DISCUSSION

**Figure 3.**
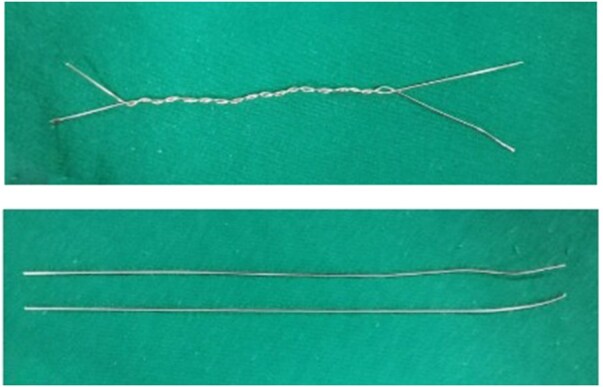
The Method of Using Wire Sutures for Bar Fixation (The Wire Sutures Used for Bar Stabilization Are Applied in a Double-Row and Straight Configuration. Twisting the Wire Sutures around Each Other Reduces Their Strength and Increases the Risk of Breakage).

The Abramson procedure is a treatment for PC deformity in selected patients, with low morbidity and excellent cosmetic results.^10–12^ However, while not as frequent as in open surgical interventions, there is still a possibility of complications. In the literature, the complication rate is reported as 34.9%, while in our study, the number of early complications requiring additional treatment or surgical intervention was 5 (5.8%), and the rate of late complications was quite low at 8.1%.^13^ Wire fracture is a common postoperative complication in this procedure, with reported incidences ranging from 5.2% to 16.6%.^10^^,^^14^^,^^15^ In Lee et al’s report, the rate is as high as 100%.^16^ Wire fracture and the resulting displacement of the bar are the most frequent causes of reoperation. In our study 3.5% rate of wire fracture was reported, which is significantly lower than the values found in the literature. The number of cases re-operated by us was 4 (4.7%). In our clinic, the wire sutures used for stabilizer fixation, which were previously twisted around each other, are now placed in 2 parallel rows in a straight fashion (**Figure 3**). This increases the resistance of the wire to breakage, and wire fractures are less common in our clinic. One of the cases underwent operation due to bar displacement, 2 cases underwent wound debridement, and 1 case underwent operation due to the need for Nuss due to overcorrection after bar removal.

In the literature, the removal of the bar is recommended between 24 and 36 months postoperatively.^17–20^ In our cases, the bar removal was most commonly performed between 25 and 36 months, aligning with the timing recommended in the literature (40 patients, 46.5%) (**Table 3**). The timing of bar removal was planned based on the patient’s age and the degree of deformity correction.

Questionnaires were developed to assess patient satisfaction following surgical correction in patients with PE. The new single-step questionnaire (SSQ) developed by Krasopoulos et al is one of the most widely accepted questionnaires for assessing the quality of life and general satisfaction in young males who underwent the Nuss procedure.^21^^,^^22^ In Krasopoulos et al’s study, the “low satisfaction cut-off value” was calculated as 52.8, by taking average of the 30% lower scores. Patients with a total score <52.8 were considered to have a low satisfaction level. However, there is no standardized satisfaction assessment questionnaire specifically designed for PC in the literature. Some studies have used modified versions of the questionnaires developed for Nuss in evaluating satisfaction in patients who underwent surgery for PC.^23^ In our study, the single-step questionnaire was revised according to the Abramson procedure applied to PC cases and used for satisfaction assessment. The total score for each patient included in the study ranged from 70 to 92, with an average value of 88.56. The average score of the lowest 10 scores (<77) was considered the “low satisfaction cut-off value” (poor satisfaction cut-off point) at 73.8. Only 5 patients (5.81%) were found to have a low satisfaction level.

## CONCLUSION

The Abramson procedure is frequently preferred in our clinic for the treatment of PC deformity in selected patients due to its low morbidity, excellent cosmetic outcomes, and high patient satisfaction. We recommend the use of the SSQ questionnaire, as it is easy to apply and analyse for assessing patient satisfaction. We suggest that further studies are needed to standardize the low satisfaction cut-off value.

## Data Availability

The data underlying this article will be shared on reasonable request to the corresponding author.
